# COVID-19 Patient with Severe Comorbidity in Multimodal Acute Care Setting with Non-Invasive Medical Ventilation: A Clinical Outcome Report

**DOI:** 10.3390/clinpract11010013

**Published:** 2021-02-03

**Authors:** Tobias Romeyke, Elisabeth Noehammer, Harald Stummer

**Affiliations:** 1 Institute for Management and Economics in Health Care, UMIT—University of Health Sciences, Medical Informatics and Technology, 6060 Hall in Tirol, Austria; elisabeth.noehammer@umit.at (E.N.); harald.stummer@umit.at (H.S.); 2 Waldhausklinik, Acute Hospital for Internal Medicine, Pain Therapy, Complementary and Individualized Patient Centred Medicine, 86391 Deuringen, Germany

**Keywords:** COVID-19, SARS-CoV-2, chronic disease, comorbidity, diabetes, chronic heart disease, non-invasive medical ventilation, pain, holistic care, Germany

## Abstract

The virus that causes COVID-19 is rapidly spreading across the globe. Elderly patients with multiple pre-existing conditions are at a higher risk. This case study describes acute inpatient treatment of a COVID-19 patient with uncontrolled diabetes mellitus, kidney complications, heart failure, chronic pain, depression, and other comorbidities in an isolation ward without mechanical ventilation.

## 1. Introduction

The novel disease severe acute respiratory syndrome coronavirus type 2 (SARS-CoV-2) was first reported in December 2019 in Wuhan, Hubei Province, China, and has since become a global pandemic and major public health threat [1]. To limit the surge of infection rates via travel, etc., many international and national regulations were put into place [2]. The virus spreads extremely fast and the clinical course often varies widely [3]. Health systems worldwide are still facing unprecedented needs of care of severe cases [4]. Protecting Intensive Care Unit (ICU) resources required by about 5% of the cases [3] has thus emerged as a major concern [5] reacted to, for example, via national or regional lockdowns [6]. How these can and should be lifted is being discussed [4], also due to their economic and social impact [7]. Therefore, research regarding potent medication as well as showing how ICUs might be relieved via other forms of treatment is vital. This study is on the latter, putting a specific focus on older patients with comorbidities, who are among the expressed risk groups for SARS-CoV-2 [8].

### 1.1. Background Information and Taxonomy

Coronaviruses are very common among mammals and birds [2] and belong to the realm Riboviria, order Nidovirales, suborder Cornidovirineae, and family Coronaviridae. The latter has two subfamilies, one of them Orthocoronavirinae, which includes four genera: Alpha-, Beta-, Gamma-, and Deltacoronavirus (Coronaviridae Study Group of the International Committee on Taxonomy of Viruses, 2020).

Via homologous recombination, coronaviruses can easily increase their spectrum of hosts even to other species [9]. There are seven known species of coronaviruses that are pathogenetic for humans (HCoV). These belong to the genera Alpha- (HCoV-NL63, HCoV-229E) and Beta-Coronavirus (HCoV-HKU1, HCoV-OC43, SARS-CoV, MERS-CoV, SARS-CoV-2). Four of these species were already prevalent on a global level (HCoV-HKU1, HCoV-229E, HCoV-NL63, and HCoV-OC43), mostly resulting in mild colds. However, severe pneumonias are possible in infants as well as elderly or immunosuppressed individuals [10].

SARS-CoV-2 is a new coronavirus (Genus: Betacoronavirus, Subgenus: Sarbecovirus) that was identified in early 2020 as the cause of the respiratory coronavirus disease 2019 (COVID-19) illness (Coronaviridae Study Group of the International Committee on Taxonomy of Viruses, 2020). SARS-CoV, MERS-CoV, and SARS-CoV-2 were only recently transmitted from animals to humans [11,12,13], with some studies suggesting a snake–human transmission for SARS-CoV-2, and others proposing bats as the culprit [2]. Infections with SARS-CoV, MERS-CoV, and SARS-CoV-2 can lead to severe illnesses with potentially fatal outcomes [2]. As human–animal transmissions of SARS-CoV-2 are possible as well, a One Health approach is advised [12].

### 1.2. Burden of Disease

A lot of research is currently being done on questions regarding mutations of SARS-CoV-2 and their virulence, transmissibility, and immunogenicity. Alm et al. [14], for example, discussed the temporal and special distribution dynamics of SARS-CoV-2 genetic clades in Europe. According to the Robert Koch Institute (RKI), in Germany alone, a total of 1,719,737 people were infected and 33,071 deaths were reported on 31 December 2020 for that year (Robert Koch Institute (RKI), 2020). The number and cumulative incidence (per 100,000 population) of laboratory-confirmed COVID-19 cases and deaths for each federal state electronically reported to RKI, Germany (31 December 2020, 12:00 a.m.), are shown in Table 1.

To date, many cases have been asymptomatic. Others have suffered from severe pneumonia, causing lung failure and death. Common symptoms include fever and cough [15,16]; however, impaired sense of taste and smell has also been reported [17]. Severe cases of COVID-19 infections resemble the clinical symptoms of endothelial dysfunction, which might even be a common denominator [18]. However, severe cases have also been reported in patients without known pre-existing conditions [19]. According to Chinese studies, the median age of Chinese patients is 51. Patients under the age of 20 are rarely affected [20]. A similar picture emerges in Germany, where the median age also is 50.

According to the RKI, inpatient treatment in Germany as of 31 December 2020 was as follows (Table 2):

Based on RKI data, patients with common pre-existing conditions are at a higher risk.

Patients with chronic lung diseases, diseases of the cardiovascular system, diabetes mellitus, or malignant neoplasms are often affected.

Long-term complications, described in the literature, are based on previous studies on SARS. They include impaired quality of life [21] and restricted lung function [22], but also post-traumatic stress disorders, depression, and anxiety disorders [23].

## 2. Aims

Our aim was to gain further insights into the clinical presentation of COVID-19 in multimorbid patients with cardiovascular diseases and uncontrolled diabetes mellitus, kidney complications, pain disorders, and urticaria. We also analyzed other comorbidities, as well as the application of conservative treatment methods without mechanical ventilation, taking into account patient experience.

## 3. Methods

This is a retrospective, single-case study reported in April 2020. The case was chosen as the patient refused ICU treatment and is based on medical records from an acute hospital in Bavaria, one of the most affected areas in Germany. The patient requested not to receive intensive care. The corresponding advance directives of the patient are available. His comorbidities were diagnosed by specialists. The treatment was provided by an interdisciplinary team comprising specialists in general internal medicine; physicians specialized in nephrology, with long-standing experience in the treatment of multimorbid patients and patients with pain disorders; holistically trained nurses; physiotherapists; and massage therapists. The patient consented in writing to the processing of his medical records.

Pain intensity was assessed with the help of the Visual Analogue Scale [24], and physical functions with the Hannover Functional Questionnaire (FFbH).

The FFbH was selected because scientific studies validate its methodical application in diseases of the musculoskeletal system [25].

## 4. Case

### 4.1. Diagnoses Backed up by Medical Specialists

Laboratory findings are shown in Table 3; chronic and acute conditions are highlighted (in bold for chronic and bold and italics for acute conditions).

Type 2 diabetes mellitus derailed with diabetic nephropathy

Acute bronchitis in COVID (SARS-CoV2 positive)

Anthracofibrosis

Progressive aortic stenosis

Pulmonary hypertension

symptomatic hyperuricemia

Chronic renal failure CKD 4

Other spondylosis with radiculopathy: lumbar region with secured neuroforamen stenosis on the right L4/L5

Gonarthrosis on both sides

Knee replacement on the right

Gastrointestinal bleeding 09/2016

Chronic heart failure NYHA (New York Heart Association) 2

Hypertriglyceridemia

Fall tendency with difficulty walking

Urinary incontinence

The multimorbid patient (84 years old) was hospitalized because his general condition was deteriorating. He reported fatigue and feeling unwell. The patient complained of general weakness and worsening drowsiness in the last 15 days. He was tired and listless. Prior to that, he had had fever up to 38 °C and a dry cough. His fever had been dropping for about 3–4 days before he was admitted to the hospital. He had been coughing mainly during the day. His throat had also been uncomfortably sore, and his appetite had decreased. Clinically, the patient was dehydrated on admission. However, he reported that he had been drinking sufficient amounts of fluids.

The patient had not been taking antibiotics earlier on. He had had no sniffles, and his sense of taste had not changed. One of his relatives had a cold, and another one had pneumonia.

The patient presented with fissured tongue and dry skin. A PCR test for SARS-CoV-2 had not been performed in advance.

The patient had had insulin-dependent type 2 diabetes mellitus for several years. In the previous 2 weeks, his blood glucose values had indicated hyperglycemia. His ICT insulin regimen had been adjusted about 5–6 months before (Levemir (Insulin Detemir) 26 IU at 10 pm and Apidra (Antidiabetic drug) with meals).

One week before admission, the patient had consulted a dermatologist for acute urticaria of unknown etiology. According to the patient’s medical history, he has had no known allergies. The patient received 100 mg Solu-Decortin (Natrium (prednisolon-21-succinat) and 8 mg IV Dimetinden (H1 antihistamines). The dermatologist prescribed a cortisone pulse therapy with 10 mg prednisolone in the morning for 5 days, and 10 mg/day of cetirizine. The patient’s blood glucose levels rose significantly under cortisone therapy and infection. He had hypoglycemia early in the morning and hyperglycemia up to 500–600 mg% during the day. Hypoglycemia caused the patient to sweat profusely. He did not report nausea, vomiting, or diarrhea. He reported having spent the previous week mostly in bed with fatigue and listlessness.

The patient has also had chronic NYHA 2 heart failure for several years. In the previous 4 days, he had noticed dyspnea, even on light exertion, when performing day-to-day activities at home.

On admission, the patient also complained about immobilizing knee pain. He had had a known chronic pain syndrome with back and joint pain. His back pain had been treated with pregabalin (Lyrica^®^) and metamizole (dipyrone) for several years. He had been taking 10 mg Doxepin at night to calm down and to relieve anxiety.

Self-reported history: symptoms of an upper respiratory tract infection for about 10 days, which had been treated for 2–3 days with acetylcysteine on an outpatient basis. Pulmonary symptoms: low-grade anthracofibrosis, which had been diagnosed two years before. Cortisone pulse therapy for spontaneous urticaria, with the resulting hyperglycemia, one day prior to the admission.

History of the autonomic nervous system: somnolence. No nicotine. Appetite significantly reduced. No known allergies. Voiding normal; nocturia 2x. Bowel movements: constipation. Height: 180 cm. Weight: 83 kg.

### 4.2. Treatment, Clinical Course, and Results

The patient was admitted to the hospital with a pronounced deterioration of his general condition. He was feeling unwell, was dehydrated, and complained of fatigue. He had had fever and a severe dry cough for 3–4 days before admission. At the time of admission, the patient’s physical functions were severely limited with a score of 8% (Hannover Functional Questionnaire). The standard value is 77%. On the day of the admission, the patient was tested for SARS-CoV-2 RNA due to his general condition and his self-reported medical history. He was then isolated according to the requirements of procedure 8–98 g.01.

The initial test result for SARS-CoV-2 RNA on April 6th was negative. In line with the guidelines, the patient was tested again on the second day after admission (thus, 7 April), following World Health Organization protocols (WHO 13.03.20). For both tests, a reverse transcription polymerase chain reaction test was administered. On 7 April, the test result was positive. The patient was reported to the relevant Health Department, and category A contact persons, who in this case were relatives who had had direct contact with the patient, were advised to self-isolate.

Immediately after the admission, we started an empirical IV treatment with Sterofundin and Cernevit (Multivitamin supplement). We carefully restored the patient’s fluid balance and started his mobilization early on. The patient was treated with frequent sessions of physiotherapy and manual therapy. He also received daily massage sessions with Vibrax and Tri-Flow therapy to stimulate his breathing. We performed an inhalation therapy with PARI BOY NaCl 0.9% (1 ampoule of Atrovent (Ipratropiumbromid), 15 drops, and Salbutamol (β₂-Sympathomimetikum), 15 drops, 3 times a day), as well as frequent rubbing with aconite oil to stimulate breathing. We also applied oxygen therapy since the patient’s spontaneous oxygen saturation was reduced to 88% on admission. The patient was administered O^2^ at 2 L/min. He did not require supplementary oxygen afterwards, and his respiratory function stabilized. His cough continued to abate, and his breathing became easier with time.

The symptoms of the patient were markedly relieved following frequent mobilization measures and breathing exercises. His dyspnea on light exertion was in remission. His inflammation parameters, which had been elevated initially, also fell over time. Initially, the patient’s renal function parameters had been out of range due to dehydration. They markedly improved over the course of the empirical IV therapy. Blood platelet count was Tsd/µL 337 at admission and 266 at discharge.

His blood glucose levels were highly elevated, and he had hyperglycemia at 405 mg% (equaling 22.48 mmol/L; thus, a reduction of 295 mg/dL would be required to reach normal levels) in the context of his type 2 diabetes mellitus, which had been known for years. The intensive conventional insulin therapy was optimized over time under close blood sugar monitoring, which was performed daily at 07:00 a.m., 11:00 a.m., 02:00 p.m., 05:00 p.m., 10:00 p.m., and 02:00 a.m. (with values ˃ 300 mg%, we performed the measurements every half an hour). We set the long-acting insulin (detemir) to 24 IU, and the patient was taking the rapid-acting insulin (apidra) with meals at increased doses. In terms of nutritional measures, the patient was following a special dietary regimen. His blood sugar levels were satisfactory on discharge.

The isolated urticaria of unclear etiology may have been caused by analgesia with metamizole (Novalgin^®^), which the patient had been using for weeks. We changed the analgesia to naproxen ((S)-2-(6-Methoxy-2-naphthyl) propionsäure) with a dosage of 250 mg (2–3 times a day as required). The patient also received treatment with alpha lipoic acid at 600 mg/day for suspected diabetic polyneuropathy. His pain, which had been between 6 and 7/10 on the Visual Analogue Scale on admission, was also alleviated thanks to all the therapeutic measures.

After the eight-day hospitalization with frequent application of the above-mentioned measures and implementation of the complex treatment OPS 8–98 g.01 (Table 4), the patient was discharged into home isolation (quarantine). There were no complications. Thus, the patient’s primary care giver (daughter) was asked upon discharge to call immediately in case of a deterioration in the patient’s condition. This has not happened so far (status: January 2021).

### 4.3. Parameters on Discharge

Oxygen saturation: SpO2 95% (without O^2^ administration)

Pain intensity (Visual Analogue Scale): Reduced to 2.1/10

Physical functions: Improved with a score of 44% (Hannover Functional Questionnaire)

Blood sugar lowered to 156 mg%

Blood platelet count at Tsd/µL 266

## 5. Discussion

The overall clinical picture is one of multimorbidity in a man with a number of comorbidities, including NYHA 2 heart failure, diabetes with kidney complications, and chronic pain at status post total endoprosthesis. Elderly patients with comorbidities are among the highest risk groups for SARS-CoV-2 [8] and fatal outcomes [26]. Wang et al. [27] performed a meta-analysis and reported that “hypertension, diabetes, COPD, cardiovascular disease, and cerebrovascular disease are major risk factors for patients with COVID-19” (p. 6049), but that no such correlations could be established for malignancy, renal diseases or liver disease. As Guan et al. [28] point out, causality and interdependencies of results on COVID-19 and comorbidities may still need to be investigated in more detail. In general, Kluge et al. [3] strongly advocate for an interdisciplinary, guideline-oriented approach. Ideally, this is targeted at reducing ICU care requirement. In our study, the patient refused ICU care, but the results showing how it could be avoided may be interesting in situations with limited ICU capacity, as well.

The time from the onset of illness to hospitalization coincided with the results of Chinese studies, i.e., 7 days on average [29]. The length of the hospital stay deviated from typical results. In most studies, it was generally 14 days, and over 3 weeks in severe cases [20].

Our example demonstrates a conservative acute treatment without invasive ventilation, presenting a pragmatic approach of treating comorbidities and COVID-19 simultaneously in an elderly patient. Advanced age is associated with higher mortality, as indicated by previous studies on SARS [30]. According to a meta-analysis by Bo Li, COVID patients with cardiovascular diseases or diabetes often require intensive medical care [31]. Zhu et al. [32] report that patients with COVID-19 and diabetes type 2 showed significantly higher mortality rates, which decreased when glycemic levels were stable, making their control vital during treatment. Reducing and optimizing blood sugar levels was one of the therapeutic interventions in our case.

Patients with severe presentations of COVID-19, who are also multimorbid, often require invasive ventilation [33]. However, due to the inclusion and exclusion criteria of the 2020 meta-analysis by Li, scientifically necessary outcome measurements of the therapeutic procedures were not given sufficient consideration [31]. Studies to date have shown hardly any therapeutic outcome measurements for the application of conservative, non-invasive ventilation. According to one study, this may also be due to lower patient adherence [34]. At present, a clear outcome assessment remains to be seen [35].

Current studies point out that a parallel treatment of concomitant diseases, such as endocrine diseases, is very significant. However, it is not adequately implemented at present [36], even though diabetes mellitus and hyperglycemia may increase mortality [37]. Patients with diabetes may be more susceptible to infectious diseases [38], which may also be attributed to immune system dysfunction [39]. In his 2020 article, Gupta provides valuable recommendations for the treatment of COVID-19 patients with diabetes [40]. The application of his recommendations is virtually identical to the patient treatment at hand. The present case study is also intended to draw attention to the fact that if changes are made to treatment regimens of multimorbid patients (for example, if cortisone is administered or pain medication is changed), the patients will require a close specialist supervision, and consideration should be given to all concomitant diseases. When the general condition of elderly multimorbid patients deteriorates, they require immediate specialist care on an outpatient basis so that they may be offered the option of an immediate hospitalization. This would help avoid intensive care treatment. After examining the clinical parameters, care providers may provide a holistic treatment for all acutely exacerbated comorbidities in order to mitigate the progression of COVID-19. However, outpatient services in Germany are not working as usual at present due to the pandemic. This may be due to patients avoiding medical appointments out of fear of infection, even when they are in urgent need of treatment and would require hospitalization.

Certainly, even older patients with comorbidities suffering from severe forms of COVID-19 are reported to survive with supportive measures alone. However, Karragianides et al. (2020) show that mortality rates for older patients with comorbidities and requiring ventilation are much higher than for the respective comparison groups, suggesting that survival rates also depend on the preconditions [26]. Instruments such as the clinical frailty scale can be employed to check the latter on a global level. While it should be used with caution in a COVID-19 context [41], the score was found to be a better indicator of COVID-19 survival rates than age or comorbidities [42], especially for older patients [43]. As frailty increases with age and health issues [41], the importance of a holistic assessment and treatment plan for elderly COVID-19 patients with comorbidities rises due to their risk status.

While a case study is not designed to quantify and establish impacts of treatment as is possible in large scale studies and ideally in RCTs, there are also vital ethical concerns connected to more experimental settings. This investigation contributes by reporting on reasonable options of treatment for specific patients. Further studies on COVID-19 should focus on therapeutic procedures that include both mechanical ventilation and conservative treatment methods, while taking comorbidities and chronic care in the context of a holistic approach into account [44]. Scientific work should also consider patient experience (quality of life, sensation of pain, physical functions, mental well-being, etc.).

## Figures and Tables

**Table 1:** Cases and deaths for each federal state (RKI, Germany 31 December 20).

Federal State	Total Number of Cases	Cases/100,000 Pop	7-Day Incidence Per 100,000 Pop	Number of Deaths	Number of Deaths/100,000 Pop
Baden-Wuerttemberg	273,993	2144	133	4789	43.1
Bavaria	324,937	2476	163	6716	51.2
Berlin	96,788	2638	126	1247	34.0
Brandenburg	41,241	1635	177	937	37.2
Bremen	13,559	1990	80	194	28.5
Hamburg	36,417	1971	100	632	34.2
Hesse	136,577	2172	132	2845	45.2
Mecklenburg-Western Pomerania	11,997	746	89	171	10.6
Lower Saxony	106,789	1336	80	1967	24.6
North Rhine-Westphalia	393,185	2191	127	6552	36.5
Rhineland-Palatinate	71,993	1759	110	1428	34.9
Saarland	19,879	2014	113	432	43.8
Saxony	132,356	3250	327	3139	77.1
Saxony-Anhalt	29,200	1330	153	602	27.4
Schleswig-Holstein	24,792	854	77	425	14.6
Thuringia	42,034	1970	246	995	45.6
Total	1,719,737	2068	140	33,071	39.8

**Table 2:** Inpatient treatment. (https://www.rki.de/DE/Content/InfAZ/N/Neuartiges_Coronavirus/Situationsberichte/Dez_2020/2020-12-31-en.pdf?__blob=publication (File accessed on 31 December 20, RKI)).

	Number of Patients
Currently in ICU	5639
- thereof with invasive ventilation	3112
Discharged from ICU	50,457
- thereof deaths	13,103

**Table 3:** Laboratory findings.

Indicator	Value	Further Information/Associations with Multiple Comorbidities
Neutrophil, Tsd/µL	7.20	Norm: 1.9–6.1 Infections with bacteria, ***viruses***, fungi or parasites can increase the value.
Basophil, Tsd/µL	0.16	Norm: ≤0.08. Diseases with higher concentrations of lipides in the blood (**Diabetes mellitus**, **Nephropathien**, Myxedema) can be associated with higher levels of basophiles.
Creatine, mg/dL	2.35	Norm: ≤1.30 (for individuals over 60). In cases where the norm is surpassed, the reasons are either acute kidney failure, **chronic kidney disease** or desiccosis (lack of water, ***dehydration***).
Lymphocyte absolute Tsd/µL	1.05	
C-reactive protein (CPR), mg/L	23.05	Norm: ≤5. Elevated CRP levels are associated with bacterial and ***viral******infections***, rheumatic diseases, **coronary diseases**, heart attacks, etc.
Triglyceride, mg/dL	215	Norm: <150. Elevated values indicate metabolism disorders. Patients with **diabetes**, **kidney diseases** or overweight often have higher levels.
Urea, mg/dL	72	Norm:10–50. Higher values in the blood serum indicate a **reduced kidney function**.
Uric acid, mg/dL	7.8	Norm: ≤7. Higher levels indicate **chronic kidney diseases**, **diabetes**, lipid metabolism disorders.
Lactate dehydrogenase, U/L	294	Norm: ≤250. Higher values indicate **coronary heart diseases**, myocarditis, pericarditis, cardiac arrhythmias, skeletal muscle diseases.
TSH basal µIU/mL	0.78	
Albumin %	46.5%	Norm: 54.7–66.0. Indicates liver diseases, ***acute inflammations***, lack of protein.
Alpha-1-Globulin %	5.6%	
Alpha-2-Globulin %	14.6%	Norm: 6.8–13.7. The values are usually higher for patients with nephrotic syndrome.
Beta-Globulin %	12.7%	
Gamma globulin %	20.6%	Norm: 10.6–19.8. Gamma-Globulin contains the major part of immunoglobulins (antibodies). A higher level indicates either a late stage of ***acute inflammation*** or subacute/chronic inflammations.
Bilirubin mg/dL	0.57	
Myelocytes %	2.7%	Myelocytes are precursors of white blood cells and capable of cell division. There are no reference values as myelocytes usually only occur in bone marrow and not in the blood, or only in very small quantities. In cases where they do occur in the blood, they are either covered in the manual differential blood count or referred to with a comment. In the peripheral blood, they can indicate certain diseases like leukemia or severe inflammation.
Chronic Kidney Disease Epidemiology Collaboration mL/min	24	Norm: ≥60; lower levels indicate renal failure (degree IV); values between 15–29 indicate a severe functional **kidney damage**.

**Table 4:** Complex treatment for infection with pathogens requiring isolation OPS 8–98 g.01.

Treatment by specially instructed medical personnel, in collaboration with the hospital hygienist and/or the nurse/nurse for hospital hygiene (hygienist) under the supervision of the hospital hygienist taking into account current treatment and care standards
Conducting special investigations to determine the settlement or infection with a non-multidrug-resistant pathogen requiring isolation
Implementation of strict isolation (individual or cohort isolation) with your own sanitary area or bed chair (avoidance of cross infections). Isolation is maintained in accordance with the current guidelines of the Robert Koch Institute (RKI)
Change of bed linen, clothing, and utensils for personal care (washcloths, etc.) according to the current guidelines of the Robert Koch Institute (RKI), if necessary daily
Protective measures when entering and leaving the room (room-related protective gown, gloves, if necessary, mouth–nose protection, infiltration, evacuation, etc.)
Special hand disinfection measures before and after patient contact when dealing with spore-forming bacteria (alcoholic disinfection and hand washing)
Daily disinfection of areas close to the patient according to the current guidelines of the Robert Koch Institute (RKI), possibly several times and/or using special area disinfectants
At least daily floor disinfection and one-time final disinfection, if necessary, using special surface disinfectants
Patient and family talks (possibly also talks with caregivers) about dealing with pathogens that are not multi-resistant and require isolation
Specific measures for the treatment or eradication of the pathogen according to the current recommendations of the RKI
Perform the following actions if necessary:
Use of pathogen-specific chemotherapy drugs/antibiotics
Implementation of the diagnostic and therapeutic measures under special spatial–organizational conditions (e.g., in the patient room instead of in the functional area; if in functional areas, then with subsequent final disinfection)

## Data Availability

The data of this article are not publicly available to protect personally identifiable information. And are available for requests of the corresponding authors.
